# Adaptive redox homeostasis in cutaneous melanoma

**DOI:** 10.1016/j.redox.2020.101753

**Published:** 2020-10-08

**Authors:** Liaisan R. Arslanbaeva, Massimo M. Santoro

**Affiliations:** a Department of Biology, University of Padua, 35131, Italy; b Veneto Institute of Molecular Medicine (VIMM), Via Orus 2, 35129, Padua, Italy

**Keywords:** Cutaneous melanoma, Melanomagenesis, ROS, Redox homeostasis, Metastasis, Tumor metabolism, BRAFi-resistant melanoma, Antioxidants

## Abstract

Cutaneous melanoma is the most aggressive type of skin cancer. Although cutaneous melanoma accounts for a minority of all types of skin cancer, it causes the greatest number of skin cancer related deaths worldwide. Oxidative stress and redox homeostasis have been shown to be involved at each stage of a malignant melanocyte transformation, called melanomagenesis, as well as during drug resistance. Reactive oxygen species (ROS) play an important and diverse role that regulate many aspects of skin cell behaviors ranging from proliferation and stemness, to oxidative damage and cell death. On the other hand, antioxidants are associated with melanoma spread and metastasis. Overall, the contribution of redox homeostasis to melanoma development and progression is controversial and highly complex. The aim of this study is to examine the association between redox homeostasis and the melanomagenic process. To this purpose we are presenting what is currently known about the role of ROS in melanoma initiation and progression. In addition, we are discussing the role of antioxidant mechanisms during the spread of the disease and in cases of melanoma drug resistance. Although challenging, targeting redox homeostasis in melanoma progression remains to be a promising therapeutic approach, especially valid during melanoma drug resistance.

## 1 Introduction

Cutaneous melanoma is the deadliest form of skin cancer and is the most occurring subtype of melanoma. The number of cutaneous melanoma cases have increased over the past few decades [1]. Although cutaneous melanoma represents less than 5% of the overall cutaneous malignancies, it accounts for the majority of skin cancer deaths worldwide [2]. Despite advances in metastatic melanoma treatment and prevention of metastatic relapse, the clinical prognosis is still very poor. Thus, understanding the mechanisms of cutaneous melanoma formation, also known as melanomagenesis, come with a high level of urgency and is mandatory for the development and implementation of novel therapeutic approaches [3].

In most cases, melanomagenesis is a linear multistep tumorigenic process that starts from nevus and/or intermediate lesions and progresses into a dysplastic tumor, which then develops into invasive lesions and metastasis [4]. Cutaneous melanoma is characterized by a high level of mutational burden and a structural rearrangement with mutational signatures of ultraviolet (UV) exposure [5]. Genomic mutations in the v-raf murine sarcoma oncogene homolog B (BRAF), neuroblastoma RAS viral oncogene homolog (NRAS), and neurofibromin 1 (NF1) are involved in melanomagenesis as they alter the mitogen-activated protein kinase (MAPK) pathway [6]. Phosphatase and tensin homolog (PTEN) loss is involved in the activation of phosphoinositol 3-kinase (PI3K) pathway [7]. These pathways are responsible for cell proliferation and survival and are frequently found mutated in melanoma patients. Other pathways which are altered in cutaneous melanoma that include increased telomere maintenance, histone modification, methylation, and the alteration of cell cycle and inhibition of apoptosis with mutations in TP53 and cyclin-dependent kinase inhibitor 2A (CDKN2A) [1]. Recent analysis of melanoma exome data revealed novel melanoma genes such as protein phosphatase 6 catalytic subunit (PPP6C), Ras-related C3 botulinum toxin substrate 1(RAC1), sorting Nexin 31(SNX31), transforming acidic coiled-coil containing protein 1 (TACC1), serine/threonine kinase 19 (STK19) and AT-rich interactive domain-containing protein 2 (ARID2) [8]. Currently, the most studied and frequent genetic cause of melanoma is ascribed to BRAF^V600E^, which is present in 50% of melanomas and responsible for an increased proliferation and the metabolic reprogramming of melanoma cells [[9], [10], [11], [12], [13]].

Recent observations suggest that a major cause of melanomagenesis is related to the increase of ROS level, oxidative stress, and redox imbalance [[14], [15], [16], [17], [18], [19]]. A growing amount of evidence shows that an increase in ROS levels contributes to the entire process of melanoma formation. In fact, epidermal melanocytes are vulnerable to oxidative stress due to the production of ROS that occurs during melanin biosynthesis and ultraviolet A (UVA) radiation [20,21]. The dramatic increase of ROS levels and oxidative stress that melanocytes are not able to properly counteract, leads to DNA and lipid damage, with consequential induction of DNA reparation/apoptosis or of the generation of tumor-initiating cells [15,16]. Epidermal melanoma cells maintain redox homeostasis with distinct changes in its bioenergetic metabolism in response to oxidative stress. Therefore, redox homeostasis (balancing oxidative stress and antioxidant response) is an underlying mechanism that contributes to melanoma initiation and is linked to the complete melanomagenesis process [22]. Importantly, an increased ROS level plays the role not only in melanoma initiation and promotion, but also in melanoma resistance [23]. The deleterious effect of ROS and redox imbalance can be controlled through the use of different ROS and redox modulators that are responsible for the supporting antioxidant capacity of the cells. This can be achieved by melanoma cells through different mechanisms that involve metabolic pathways such as pentose phosphate pathway [24], serine biosynthesis [25], 1-carbon metabolism [26], mitochondrial metabolism [11,27] and lipogenesis [28]. Here we describe currently known cellular mechanisms that are involved in redox balancing and their regulation by different ROS sensors in metastatic and drug-resistant melanoma cells. We believe that by understanding redox homeostasis in melanomagenesis and melanoma drug resistance we would open up a new hunting ground for targets in the development of combined therapy.

## 2 Role of ROS and UV radiation during melanoma formation and progression

### 2.1 Role of UVA radiation in ROS generation

**Fig. 1:**
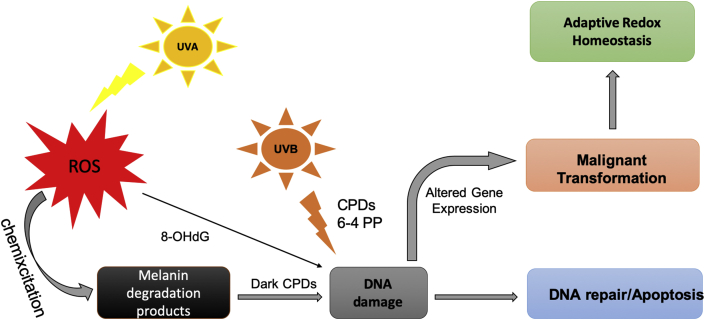
Scheme of malignant transformation of melanocytes into melanoma by ROS and DNA damage. Melanocytes are susceptible to oxidative stress due to UVR and melanin biosynthesis which involves ROS generation. UV light induces DNA damage. UVB radiation directly induces DNA damage with creation of CPDs and 6–4 PP products (described in Refs. [20]). UVA radiation interacts with cellular components of melanocytes in a process called chemiexcitation with consequent ROS generation, production of oxidized melanin products and DNA damage. DNA mutations must be repaired by DNA-repairing machine or cells will die by apoptosis. If mutations are not repaired they can induce melanomagenesis and therefore alter redox state to combat oxidative stress. UVA, ultraviolet A; UVB, ultraviolet B; ROS, reactive oxygen species; 8-OHdG, 8- hydroxy-2′-deoxyguanosine; CPDs, cyclobutane pyrimidine dimers. 6–4 PP, pyrimidine (6–4) pyrimidone photoproducts (6–4 PP).

The WHO (World Health Organization) continues to report that ozone levels are diminishing, which allows for more solar ultraviolet radiation (UVR) to reach the Earth's surface. It is estimated that a 10% decrease in ozone levels will increase the amount of melanoma skin cancer cases by an additional 4500 (www.who.int/news-room/q-a-detail/ultraviolet-(uv)-radiation-and-skin-cancer). UVR is the main exogenous factor responsible for DNA damage and ROS production involved in melanoma initiation and progression. Ultraviolet radiation induces DNA damage in different ways: a) ultraviolet B (UVB) induces the formation of cyclobutane pyrimidine dimers (CPDs) and 6-4 photoproducts (6–4 PP), which are responsible for C→T transitions; b) UVA is responsible for the ROS production and following 8-hydroxy-2' -deoxyguanosine (8OhdG)-mediated DNA damages [20]. The mutagenic properties of UVR drive the initiation of melanoma are illustrated in Fig. 1. Endogenous chromophores (flavins, nicotinamide adenine dinucleotide phosphate (NADPH), urocanic acid) absorb UVA in melanocytes and, then, by means of photosensitization mechanisms produce ROS and consequent DNA damage [14,29].

### 2.2 Role of melanin pigments in ROS generation

Human melanocytes synthesize eumelanin and pheomelanin pigments, which have different photoprotective roles. It is generally accepted that eumelanin protects the skin against photodamage, instead pheomelanin does not have a UV-protective role [30]. In contrast another study shows that eumelanin has a pro-oxidant melanoma-inducing function. Using mammalian models, it has been shown that the UVB-mediated melanoma is pigment-independent, instead UVA-mediated melanoma requires oxidized eumelanin and is associated with oxidative DNA damage [21].

Another pigment, pheomelanin, which is responsible for red hair skin phenotype, has been associated with oxidative stress-dependent melanoma induction. A study was done on colored mouse hair, where it was shown that pheomelanin depletes glutathione (GSH) only upon UVA radiation [31]. An opposite report showed that the purified pheomelanin oxidizes GSH and NAD(P)H in solution in UV-independent manner [32]. In addition, pheomelanin pigment may induces oxidative stress, lipid damage, and consequent melanoma induction in murine model with BRAF^V600E^ mutation and melanocortin 1 receptor (MC1R) inactivation in melanocytes (to mimic red skin phenotype). Indeed, when crossed with an albino allele which ablates the biosynthesis of pheomelanin, it was protective for melanoma initiation [15]. The role of pheomelanin in the formation of DNA photoproducts, named “dark” CPDs, and double DNA breaks was shown in mouse models, with observed DNA damage after a cessation of UV exposure. It proves that DNA damage is the result of the process “chemiexcitation”. This was first shown by authors stating that melanin fragments are able to migrate to the nuclei of cells. And secondly that UV-induced ROS excite electrons in melanin fragments (especially pheomelanin), in which its state is changed from singlet to triplet. Melanin fragments, which are present in the nuclei, transfers the energy of UV photon from their excited electron to DNA with the creation of CPDs products, named “dark” CPDs, because they were observed after blocking of UVR [16]. In the end we can conclude that the role of melanin pigments in UVR protection, particularly the role of eumelanin, is far to be been understood and it should be carefully further investigated.

## 3 Balancing oxidative stress by increasing antioxidant power in melanomagenesis

Cancer cells develop adaptive responses against oxidative stress by upregulating their antioxidant scavenging capacity [33]. Redox homeostasis is regulated by different transcription factors and modulators, generally by nuclear factor erythroid 2-related factor 2 (Nrf2), which controls ROS levels by upregulating antioxidant pathways that produce NADPH and GSH [34]. In general, antioxidant systems of melanoma cells buffer the increased levels of ROS through the direct and non-direct scavenging of ROS. Superoxide dismutases (SODs), catalase (CAT) and glutathione peroxidases (GPx) directly convert ROS into water and oxygen. If ROS evade the direct scavenging and start to oxidize proteins, lipids and DNA, the thioredoxin (Trx) and glutaredoxin (Grx) systems reduce the damage by coupling reactions. Both systems are dependent on NADPH that derives from the oxidative branch of the pentose phosphate pathway (PPP) and act to to reduce oxidized Trx and oxidized glutathione (GSSG). Glutathione peroxidases such GPx4 use reduced glutathione (GSH) to reduce lipid peroxides [35,36]. Interestingly, it has been suggested that the redox capacity of melanoma could be on a continuum: low capacity (normal skin), moderate capacity (drug-sensitive melanomas), and high capacity (drug-insensitive melanomas) [37].

### 3.1 ROS modulators at transcriptional sites

During melanoma formation and progression, it has been found that a number of genes are responsible for ROS detoxification. They are upregulated by various transcription factors as well as ROS sensors, and in turn, they act by buffering ROS-mediated oxidative stress and maintaining redox homeostasis in cancer cells [33]. Here we focus on the most important ROS modulators involved in antioxidant responses in melanoma, such as transcription factor nuclear factor erythroid 2-related factor 2 (NRF2), transcription factor kruppel-like factor 9 (KLF9), signaling axis of microphtalmia-associated transcription factor (MITF), peroxisome proliferator-activated receptor ϒ coactivator 1α (PGC1α), transcription factor forkhead box protein M1 (FOXM1) and signaling pathway of prolyl hydroxylase domain protein 2 (PHD2) and hypoxia-inducible factors (HIF) and signaling axis of Ca^2+^/calcineurin and nuclear factor of activated T cells (NFAT) nuclear factor of activated T cells (NFAT).

The transcription activator NRF2 is a master regulator of the antioxidant response and acts by upregulating antioxidant proteins and detoxifying enzymes [38]. Nrf2 has a protective role in UVR-induced oxidative stress, DNA damage, and apoptosis of melanocytes [39]. On the other hand, it may play a role in tumor-promotion [40]. High levels of Nrf2 have been observed in different cancer types and also in melanoma. Elevated Nrf2 expression in melanoma is correlated with a deeper Breslow index (that describes how deeply the melanoma invades into the skin), invasive phenotype, nodular growth, and poor survival [38]. It was shown that co-treatment of brusatol, a potent Nrf2 inhibitor, and UVA, a ROS-inducer, suppressed melanoma cell proliferation *in vitro* and *in vivo*, and led to apoptosis [41].

KLF9 is a transcription factor which belongs to the evolutionary conserved family of KLF transcription regulators. Klf9 alters the expression of the several genes involved in ROS metabolism. Although it has been implicated in different type of cancers [42], its role in melanoma has only recently been addressed [18]. By comparing BRAF^V600E^ *vs* BRAF^V600E^/PTEN^-/-^ mouse melanoma models, Bagati and colleagues elegantly showed that Klf9 deficiency does not affect primary tumor growth but it does promote melanoma metastasis. Also, KLF9 levels decrease during melanoma progression supporting a tumor suppressor function for KLF9-dependent ROS signaling at advanced stages of melanoma progression. These data again support a dynamic role of ROS in cancer initiation and progression [43].

The role of MITF-PGC1α axis was shown to be responsible for mitochondrial biogenesis leading to consequent changes in ROS level in melanoma cells. The MITF transcription factor regulates the development of cells from neural crest to melanocytes and is critical for melanomagenesis [44]. MITF drives the overexpression of PGC1α, a transcription coactivator, that promotes mitochondrial biogenesis and OXPHOS (oxidative phosphorylation) [11]. However, the role of PGC1α in melanomagenesis remains debatable. Some studies show that PGC1α is overexpressed and has ROS-detoxifying, antioxidant (increasing of GSH) and tumor-promoting role [45]. Also, Vazquez et al. showed that MITF-upregulated PGC1α positive melanoma cells have increased ROS detoxification, instead PGC1α negative cells display aerobic glycolysis phenotype and are sensitive to ROS-inducing drugs [27]. On the other hand, different studies showed that PGC1α suppresses melanoma metastasis. Melanomas with activation of the mutated BRAF have suppressed levels of MITF and PGC1α and decreased oxidative metabolism [11]. It was reported that overexpressed PGC1α supports mitochondrial metabolism and suppressed melanoma metastasis. The level of PGC1α was inversely correlated with vertical growth in human melanoma [46]. Thus, it is still important to clarify the role of mitochondrial metabolism in ROS generation or ROS detoxification.

FOXM1 is a proliferation-associated transcription factor and an essential regulator of oxidative stress that is expressed during cell cycle and it was shown to be overexpressed in melanoma [47,48]. The role of FOXM1 in regulation of ROS was shown in human fibroblasts by Park et al. Increased ROS level induced an expression of FOXM1, which stimulates the expression of manganese superoxide dismutase (MnSOD), CAT and Prx3 [49]. The tumor cells overexpressing FOXM1 are resistant to apoptosis or senescence caused by oxidative stress [50]. These data suggest that oncogene-induced ROS accumulation activates FOXM1 to function as an important antioxidant regulator in melanoma cells.

The role of PHD2 in the regulation of hypoxia-inducible factor (HIF) and PI3K pathways in melanoma initiation and progression was demonstrated by Liu et al. PHD2 protein, a master “oxygen” sensor, is significantly reduced in human melanoma samples and low PHD2 expression is associated with poor clinical outcome. The role of “oxygen sensor” PHD2 in protection from melanoma initiation by regulation of HIF1α and HIF2α subunits was shown on recently generated mouse model Tyr:CreER; PHD2^lox/lox^;BRAF^V600E^ possessing melanocyte-specific BRAF^V600E^ and PHD2 loss. Deletion of PHD2 in combination with expression of BRAF^V600E^ in melanocytes were enough to trigger melanoma initiation, and the development of melanoma and lymph node metastasis in mouse models. Melanocyte-specific loss of PHD2 leads to the stabilization of HIF1α and HIF2α and an activated PI3K signaling pathway, which is important for cell survival and proliferation. Authors also reported recent studies that PHD2 can directly inactivate AKT protein, part of PI3K signaling pathway, by the hydroxylation of two proline residues. These data show that PHD2 is responsible for suppressing melanomagenesis by destabilizing HIF and suppressing PI3K signaling pathways in melanocytes [51].

One last critical transcriptional pathway in melanoma progression is represented by the Ca^2+^/calcineurin–Nuclear Factor of Activated T cells (NFAT) signaling cascade. Melanoma cells express several members of the Ca^2+^/calcineurin-regulated NFAT family of transcription factors, that lead to melanoma survival via interleukin-8 (IL8) and metalloproteinase-3 (MMP-3) expression [52]. Therapeutic blockade of calcineurin/NFAT pathways not only induced apoptosis of melanoma cells, it also enhanced the antitumor effects of target-specific drugs, such as MEK or BRAF inhibitors [53]. It has been shown that oxidative stress, induced by mitochondrial ROS, is important to inhibit melanoma progression. Recent studies describe transcriptional factor NFAT1 as controling gene expression of mitochondrial proteins and promoting melanoma proliferation and migration. Data has been shown that thioredoxin-related transmembrane proteins 1 and 3 oxidoreductases (TMX1 and TMX3) are upregulated and localized in the mitochondria-associated membranes of the ER, and are responsible for control of mitochondrial ROS in melanoma cells [54]. Inhibition of TMXs lead to an increase of mitochondrial ROS promoting oxidation and inhibition of redox sensitive-dephosphatase calcineurin, which is responsible for activation of NFAT1 and melanoma proliferation and migration. Alltogether these data support a new TMX-ROS-NFAT siganling axis required for melanoma progression.

In conclusion, there are many different transcriptional-mediated redox-sensitive pathways in melanoma cells which could tune the antioxidant protection in response to oxidative stress.

### 3.2 Antioxidant enzymes

The functional role of canonical antioxidant enzymes in melanomagenesis is far from being completely understood. Bioinformatics’analyses showed a redundancy of Grx and Trx systems in tissues and the upregulation of both in melanoma [55]. The upregulation of CAT, SODs, GPx and glutamate-l-cysteine ligase catalytic subunit (GCLC) in human melanoma biopsies and a large set of melanoma cell lines was demonstrated in a systematic review [19]. Tissue microarrays containing human nevi and melanomas have high levels of thioredoxin reductase 1 (TR1), which significantly correlates with melanoma progression. Simultaneous targeting of TR1 and glycolysis suppresses growth of melanoma cells *in vitro* and *in vivo* [56].

The important role of antioxidant response during melanomagenesis was shown by the overexpression of heme-oxygenase-1 (HO-1), a Nrf2 target. HO-1 is known to be essential for heme and iron homeostasis and responsible to metabolize heme, generator of oxidative stress, into bilirubin/biliverdin, CO and ferrous iron. HO-1 was shown to be upregulated in B16F10 murine melanoma cells and *in vivo* melanoma tumor models. Cells with overexpressed HO-1 had increased proliferation rate, improved resistance to H_2_O_2_-induced oxidative stress and angiogenic activity compared to controls. Tumor-bearing mice had an augmented metastasis and decreased survival [57]. The role of other direct enzymatic ROS scavengers such as glutathione *S*-transferase (GST), which catalyzes the conjugation of GSH to a variety of endogenous and exogenous electrophilic compounds, and SOD was shown in melanoma samples in direct correlation to the Clark Level, a level of anatomical invasion of melanoma in the skin [58].

Antioxidant enzymes which protect cells from lipid peroxidation could also play the tumor-promoting role. A recent work by Morrison group showed the prooncogenic and ferroptosis protective role of acyl-Coa synthetase long-chain family member 3 (ACSL3) converts fatty acids into fatty acyl-CoA esters to incorporate into phospholipids of membrane. High level of ACSL3 was associated with poor outcome in melanoma patients. Authors identified that ACSL3 is responsible for incorporation of oleic acid in the membrane of melanoma cells in lymphnode and thus protects metastatic cells from ferroptotic cell death [59].

### 3.3 Redox buffers: GSH and NADPH

GSH and NADPH are two main cellular antioxidants, which are produced from different metabolic pathways. In normal melanocytes GSH is important to support redox homeostasis during melanin biosynthesis and for the reduction of H_2_O_2_ [60]. The cellular concentration of GSH varies from 1 to 10 mM, which allows to scavenge ROS. In melanoma cells, high GSH/GSSG ratio was shown to have an important role in metastatic progression [26,61,62]. Lomefloxacin, a fluoroquinone antibiotic, was found to deplete the endogenous GSH, inducing oxidative stress and subsequent apoptosis in the melanoma cell line COLO829. The observed pharmacologic effect of lomefloxacin makes it considerable as a drug to treat melanoma [63].

NADPH is important for the reduction of the protein-based antioxidant system and recycling of GSH and Trx. In melanoma cells the level of NADPH is regulated by Nrf2, which activates metabolic pathways (oxidative pentose phosphate pathway and one-carbon metabolism), that produce NADPH. The role of one-carbon metabolism in NADPH production in melanoma was shown recently and will be discussed later in this review.

### 3.4 Role of Coq10 antioxidant

CoQ10 (aka ubiquinone) is an important endogenous lipid antioxidant and its level decreases during skin ageing [64]. The supplementation of CoQ10 slowed down the ageing of the skin by protecting it from UV-induced ROS [65]. Deficiency of CoQ10 was observed in numerous diseases. There are few data about the role of CoQ10 in melanoma progression. A study measuring CoQ10 level in melanoma demonstrated that its level is lower in plasma of melanoma patients compared to control subjects. This increased level was associated with tumor thickness with high CoQ10 concentration in thinner tumors. In addition, it was observed that the patients with metastasis had lower CoQ10 levels than the metastasis-free patients [66]. Further studies addressing the non-mitochondrial level of CoQ10 and functional role of CoQ10-forming enzymes, such as UbiA Prenyltransferase Domain-Containing Protein 1 (UBIAD1), in the melanomagenesis are needed [67].

### 3.5 Role of exogenous antioxidants

The skin is equipped with an antioxidant network to protect itself from UV-induced oxidative stress and photoaging. In the last 20 years the use of antioxidants by cancer patients has remained a big question in medical science [68]. There are studies showing that antioxidants inhibit melanoma, as it was shown for the melanoma-inhibiting effect of antioxidant Fisetin, a plant polyphenol from the flavonoid group, during the treatment of BRAF-mutated human xenograft in mice [69,70]. However, other studies showed that the consumption of antioxidants decreases the incidence of cutaneous melanoma, but does not show any strong or significant association with melanoma suppression [71]. And a third group of studies showed that antioxidants promote melanoma metastasis. It was already suggested in 2005 that the use of antioxidant dietary supplements during chemotherapy or radiotherapy, as for any unproven agent, may be harmful [72]. The antioxidants N-acetyl-cysteine (NAC) and Trolox (soluble vitamin E analog) were shown to increase the metastatic properties of human melanoma cells [73]. In the end the tumor-suppressing role of exogenous antioxidants in melanoma treatment has not been proven yet. New studies are required to clarify their role. At this moment it is possible to conclude that exogenous antioxidants prevent skin cancer initiation due to scavenging of radicals, but also they most likely assist in melanoma metastasis.

## 4 Role of organellar comunication and associated redox regulation in melanomagenesis

Organelles morphology and intracellualar contact sites play key roles in healthy *vs* tumor tissue. Recent data have shown that healthy tissues have a consistent mitochondrial morphology and organization as well as protein expression, while those patterns disappear in skin cancer [74]. Interesting multifoton confocal microscopy work have proven that endogenous fluorescence of bound form of nicotinamide adenine dinucleotide phosphate (NADH) could be used as a marker for mitochondrial clustering and during the redox state of cutaneous melanoma. In this work authors used mitochondrial clustering as a quantitative metric of mitochondrial organization, showing the ability of mitochondria to dynamically fuse (fusion) and separate (fission) to optimize energy metabolism. Melanoma does not have a feature of depth-dependent variations of mitochondrial clustering compared to healthy skin, which could be as a result of metabolic changes [74]. It was proposed that mitochondrial clustering is dependent on the state of glycolysis or OXPHOS. Cells which possess more fragmented mitochondrial phenotype rely on glycolysis, instead cells with a more extensive mitochondrial network that had switched from glycolysis to OXPHOS [[74], [75]].

Mitochondria-ER contacts are also responsible for cellular homeostasis, and also for the control of redox signaling. Mitochondria and ER are both sources of ROS, therefore ROS is diffused through the contacts between these organelles. Deleterious ROS levels affect the organellar structure and function [76]. Recently, Bogeski group showed that ROS which are generated from mitochondria-ER contacts, can affect melanoma proliferation and migration. They identified that TMX1 and TMX3 oxidoreductases, which are responsible for mitochondria-ER communication, are upregulated in human melanoma samples. TMX1/3 depletion altered both mitochondrial organization and metabolism as well as inducing oxidative stress leading to a suppression of melanoma growth [54]. Another group showed that resveratrol, dietary phenol present in numerous plants and dietary supplements, might induce oxidative stress, which led to ER stress and mitochondrial dysfunction with subsequent apoptosis of A375SM melanoma cells [77]. These findings suggest a mechanistic link between subcellular structure and redox state in cells that need further investigation in the context of melanomagenesis and progression.

## 5 The connection/link between cancer metabolism and redox homeostasis

It has been shown that melanoma cells regularly reprogram their metabolism to provide an equivalent reduction and support in antioxidant protection. There are different signaling pathways involved in suppliyng and regulation redox power in melanoma cells. In particular, it has been demonstrated that during melanomagenesis the ox-PPP, serine biosynthesis and 1-CM are responsible for NADPH and GSH production. On the other hand, monocarboxylate transporters (MCTs) and glycolytic enzymes, such as pyruvate kinase (PK)-M2 (PKM2), are also associated to redox homeostasis during melanoma initiation and progression.

### 5.1 Role of pentose phosphate pathway (PPP)

Pentose Phosphate Pathway (PPP) is primarily catabolic and serves as an alternative glucose oxidizing pathway for the generation of NADPH that can be involved in redox metabolic adaptation of melanoma. The antioxidant role of glucose-6-phosphate dehydrogenase (G6PD), the rate-limiting enzyme of PPP that catalyzed the first reaction with the production of NADPH, has been studied in an *in vitro* melanoma model. Indeed, the inhibition of G6PD sensitized malignant melanoma cells A375 to oxidative stress, decreased proliferation and induced apoptosis [78]. Another study showed that high expression of G6PD promotes melanoma growth via the signal transducer and activator of transcription 3/5 (STAT3/5) pathway in a human melanoma xenograft model [24]. The role of G6PD in cooperation with NADPH oxidase 4 (NOX4) for the support of redox homeostasis has been related to melanoma cells *in vitro*, indeed targeting both enzymes suppressed cell proliferation [79]. Hence, these studies indicate that PPP represents an essential redox metabolic pathway in melanoma and serves a pivotal role in survival and adaptation of melanoma cells.

### 5.2 Role of glycolysis

Glycolysis is a metabolic pathway converting glucose into pyruvate and lactate as final metabolites, and releasing energy to form ATP and NADH molecules. Several enzymes belonging to this metabolic pathway have been found to be associated with melanomagenesis.

Melanoma cells overexpress a redox-depending enzyme PKM2, an isoform of the pyruvate kinase, that converts phosphoenolpyruvate into pyruvate, is the last irreversible reaction of aerobic glycolysis [80]. ROS oxidizes a specific cysteine residue in PKM2, thus diverting glucose away from lactate production and towards the oxidative branch of PPP leading to increased NADPH production and thus redox homeostasis. These data provide a direct link between cancer metabolism and redox homeostasis. Melanoma cell invasion and metastasis levels were positively correlated with high PKM2 activity as well as the glycolytic capability. In addition, knockdown of PKM2 markedly attenuated the malignant phenotypes of melanoma cells including cell proliferation, invasion and metastasis *in vitro* and *in vivo*, suggesting that PKM2 is a potential therapeutic target in melanoma [80].

MCTs, particularly MCT 1 and MCT4, which enable bidirectional passive transport of lactate and related monocarboxylates, are upregulated in malignant melanoma [23,81,82]. Clinicopathological significance of MCTs was shown in metastatic samples of melanoma: MCT4 expression significantly increased in metastatic samples, and MCT1 and MCT4 were significantly associated with poor prognostic variables [82]. Lactate synthesis and export from highly glycolytic cells are necessary to remove acid and to sustain glycolysis. Lactate was, thus, considered a waste product that must be eliminated by cancer cells. Melanoma uses MCT1 to transport lactate from the circulation into the tumor with metabolites of TCA such as citrate, glutamate, and malate. Genetic studies have shown that MCT1 plays a key role in melanoma. Indeed, using PDX and mouse melanoma models, Tasdogan and colleagues showed that the inhibition of MCT1, while not altering primary tumor formation, does lead to a depletion of circulating melanoma cells and a decrease of metastasis [23]. MCT1 inhibition leads to an induction of ROS and affects the lactate import that can alter intracellular pH and the NAD+/NADH ratio, because lactate is co-transported with a proton and converts to pyruvate intracellularly, thus converting NAD+ to NADH. MCT1 inhibition significantly increased intracellular pH, strongly suggesting substantial MCT1-dependent lactate and proton import in these tumors. The increase in pH after MCT1 inhibition could reduce flux through PPP relative to glycolysis as increased pH activates the activity of phosphofructokinase and suppresses the activity of G6PD, rate-limiting enzymes in glycolysis and PPP, respectively [23]. Thus, MCTs were shown to be importnat contributors to melanoma agressiveness and they could be an attractive targets for melanoma therapy.

### 5.3 Serine biosynthesis and one-carbon metabolism (1-CM)

Serine is a precursor for the synthesis of GSH and also a major donor of one-carbon units to 1-CM. Phosphoglycerate dehydrogenase (PHGDH), the first enzyme, which diverts glucose-derived carbon into *de novo* serine synthesis pathway, was found to be amplified and overexpressed in melanoma, indicating its clinically relevant potential. Authors performed genetic studies to show that the PHGDH gene is amplified in 16% of all cancer types and 40% in melanoma. PHGDH silencing in melanoma cell lines with increased PHGDH copy number leads to the signficant growth inhibition [83]. It was shown that upregulated level of PHGDH is important for tumor initiation and promotion in melanoma mouse model TyrCreER: BRAF^V600E^; PHGDH^tetO^ in cooperation with BRAF^V600E^ mutation. Accordingly, increased dietary serine and overexpressed PHGDH promoted tumor growth in mice [25].

1-CM is responsible for the transfer of 1-carbon unit through folate intermediates, coupling the folate and the methionine cycle, which occurs both in cytosol and mitochondria. 1-CM is commonly up-regulated in tumors with a significant impact in cancer cells with the production of NADPH and GSH [84]. Piskounova et al. showed the role of 1-CM, particularly folate pathway in NADPH production in metastatic tumors. The metastatic nodules exhibited increased amounts of serine and glycine as compared to subcutaneous tumors. Successfully metastasizing melanomas have increased the dependence on NADPH-generating enzymes in the folate pathway. Folate pathway inhibition by methotrexate, or by knockdown of its key enzymes, inhibited distant metastasis without affecting the growth of primary tumors in the same mice [26]. Inhibition of 1-CM is already used as a therapeutic strategy in cancer and combined with other agents could prove useful in combating melanoma [85].

### 5.4 Mitochondrial function (Sirt3/MnSOD)

Sirt3, a major mitochondrial NAD^+^-dependent deacetylase, which upregulates the antioxidant enzyme MnSOD, is important for melanoma survival. Sirt3 was shown to be overexpressed in human melanoma cell lines and in clinical melanoma tissue samples. Depletion of Sirt3 resulted in senescence induction, while its overexpression lead to the proliferation of melanoma cells. In a xenograft mouse model, lack of Sirt3 inhibited tumor growth and improved overall survival rate [86]. It was also found that mutant p53 induces the expression of Sirt3, and subsequent MnSOD enzymatic activity. Sirt3-MnSOD axis is therefore important for cell proliferation and survival and thus can provide new therapeutic target to fight melanoma [87].

## 6 Metabolic rewiring in BRAFi-Resistant melanoma: the role of oxidative stress in drug resistance

Almost 50% of melanoma patients harbor a driver for a mutation found in the BRAF gene, therefore BRAF studies provide for a target-based therapy to control this disease [9,10]. Vemurafenib, BRAF inhibitor (BRAFi), approved by the Food and Drug Administration (FDA) in 2011, demostrated a low toxicity level and high efficiency in melanoma patients. However, patients who responded to BRAF inhibitors treatments typically developed a resistance and relapsed within 6–8 months of treatment [88]. Thereafter, MAPK/Erk kinase (MEK) inhibitors were added to BRAF inhibitors, which may have doubled the time of progression. FDA-approved BRAF and MEK inhibitors have enhanced the prognosis of patients with BRAF mutations, but this combination also lacked reliability and strength. Therefore, overcoming reduced sensitivity and acquired resistance to targeted therapy is a major goal of current melanoma research. A better understanding of the mechanisms underlying this drug resistant is therefore mandatory. Melanoma models with an acquired resistance to BRAF inhibitors, mostly to vemurafenib, became a model to study the mechanisms of drug resistance. Indeed, one intriguing cause of drug resistance is represented by redox metabolic rewiring induced by drug resistance [89].

### 6.1 The BRAF^V600E^ mutation regulates redox homeostasis

BRAF^V600E^ mutation is associated with high levels of aerobic glycolysis genes and suppresses OXPHOS in melanoma cells [11,12,27]. Mutations of BRAF are responsible for redox metabolic rewiring BRAF^V600E^ mutation leads to the switch from OXPHOS to the aerobic glycolysis, to a decrease in numbers of mitochondria and increased production of lactate [11,13,90].

While BRAF and also NRAS mutations have been shown to upregulate Nrf2, the main antioxidant regulator, the BRAF^V600E^ mutation by itself upregulated transcription factor Klf9, which sensitizes cells to oxidative stress [18]. Interestingly, it has been also shown that Nrf2 might amplify oxidative stress via induction of Klf9 [91] making the regulation of Nrf2-Klf9-mediated response in the context of tumor and melanoma progression rather unclear and controversial to the common role of Nrf2. Therefore, the role of BRAF and also NRAS mutations alone or in combination in the metabolic switch of melanoma needs to be further investigated.

Melanoma cells can develop intrinsic and functionally acquired drug resistance types, which have different mechanisms and developmental timeframes. It was shown that short-term treatment with vemurafenib suppresses glycolysis while promoting mitochondrial respiration (OXPHOS) leading to the production of mitochondrial ROS in melanoma cells [[92], [93], [94]].

Original works have described that highly proliferative melanomas leads to a slow-cycling cell subpopulation that is identifiable by the expression of the histone demethylase JARID1B. JARID1B is a member of the highly conserved family of jumonji H3K4 demethylases [95]. This population of JARID1B^high^ slow-cycling subpopulation is required for the continuation of melanoma tumor growth. Later, the same group examined whether the subpopulation of JARID1B^high^ slow-cycling melanoma cells displayed a lower drug susceptibility compared to that of the bulk of tumor cells, discovering that JARID1B promotes melanoma intrinsic drug resistance via mitochondrially controlled reprogramming and the alteration of ROS production [96]. Quantitative proteome profiling established that this slow-cycling subpopulation relies on OXPHOS, compared to that of rapidly growing melanoma cells, is instead characterized by glycolytic metabolism and low level of JARID1B. Slow-cycling cell phenotype relying on OXPHOS provided an additional protective mechanism that helped cells to survive following an initial treatment of BRAFi by extending the time needed for the cells to establish drug resistance. As a matter of fact, inhibition of mitochondrial respiration sensitized JARID1B^high^ slow-cycling melanoma cells to therapy. These results support a combined approach to fight melanoma by blending anti-cancer agents that eliminate rapidly proliferating melanoma cells with inhibitors of the drug-resistant slow-cycling subpopulation [95,96].

**Fig. 2:**
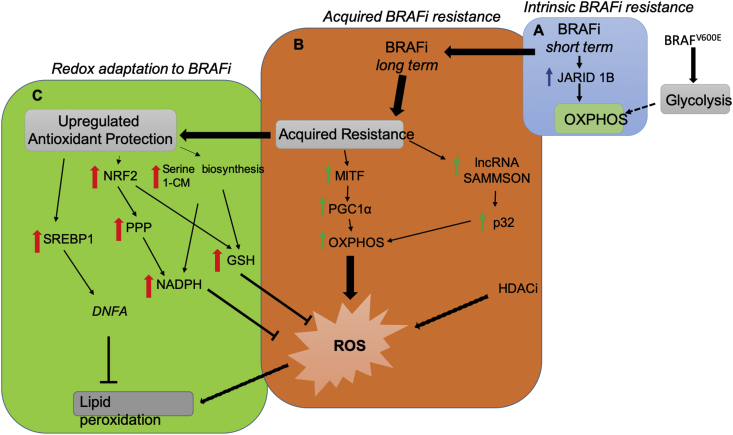
Metabolic rewiring and redox adaptation induced by BRAFi during melanoma progression. (**A**) Short term treatment with BRAF inhibitors such as vemurafenib induces the selection of pre-existing resistant subpopulation in heterogenous melanoma with overexpressed JARID1B demethylase and upregulated OXPHOS. Intrinsic short-term resistance helps melanoma cells to survive during the first BRAFi treatment and provides the time to establish an acquired long-term resistance. (**B**) Long-term BRAFi-resistant melanoma cells display high dependence on OXPHOS, which is controlled by MITF and PGC1α [27]. It was also proposed that BRAFi-resistant cells show an increase of lncRNA SAMMSON that in cooperation with mitochondrial protein p32 increases ROS level by upregulating a mitochondrial respiration phenotype [97]. Interestingly, deleterious effect of ROS could be a promising target in melanoma drug-resistant cells, as it was demonstrated by HDAC inhibitors treatments and consequent increasing of ROS level and cell death [103]. (**C**) Metabolic adaptation to BRAFi and consequently antioxidant protection is important to support cell survival of drug-resistant melanoma cells. Aberrantly activated transcription factors NRF2 upregulates PPP to produce NADPH and GSH. Activated transcription factor SREBP1 may elevate DNFA to promote lipid peroxidation [28]. Overexpressed enzymes of serine biosynthesis and 1-CM are important for melanoma BRAFi-resistant cells to supply redox equivalents to combat oxidative stress [26]. BRAFi, the v-raf murine sarcoma oncogene homolog B inhibitor; OXPHOS, oxidative phsophorilation; MITF, microphtalmia-associated transcription factor; PGC1α, peroxisome proliferator-activated receptor gamma coactivator 1-alpha; lncRNA SAMMSON, long non-coding RNA SAMMSON; HDACi, histone deacetylase inhibitor; ROS, reactive oxygen species; NRF2, nuclear factor erythroid 2-related factor 2; SREBP-1, sterol regulatory element-binding protein 1; PPP, pentose phosphate pathway; NADPH, reduced form of nicotinamide adenine dinucleotide phosphate; GSH, reduced gluthatione; 1-CM, one carbon metabolism; DNFA, *de novo* fatty acid biosynthesis.

During long term treatment of BRAFi, melanoma cells develop an acquired drug resistance with the changing of metabolic programs. It was established that there are some regulators that are responsible for the metabolic switch from aerobic glycolysis to mitochondrial respiration, subsequent increased ROS production and increased redox response (Fig. 2). Drug-resistant melanoma cells possess the mitochondrial respiration phenotype and suppressed aerobic glycolysis, which is opposite to the situation with drug-naïve melanoma cells. There are two axes that are described to be responsible for the upregulation of OXPHOS in drug-resistant cells. The first one is the upregulated axis MITF-PGC1α, which is well established in drug-resistant melanoma cells, and is responsible for activated OXPHOS and unavoidable ROS generation. The second axis long non-coding RNA (lncRNA) SAMMSON-p32 was described by Marine lab [97]. SAMMSON, long non-coding RNA, was shown to be expressed in more than 90% of melanomas and co-amplified with MITF in 10% of melanomas, because of downstream coding position. It was shown to be important for melanoma survival by interaction with p32, protein required for OXPHOS and mitochondrial integrity. It should be noted the contradiction with the data about MITF-PGC1α axis in melanoma drug-naïve cells. As it was described above, these data are controversial. Some studies show the MITF-PGC1α axis is activated in melanoma drug-naïve cells, it induces OXPHOS and makes cells resistant to oxidative stress [45]. Other studies showed that the MITF-PGC1α axis is downregulated in melanoma cells and has a tumor-suppressing role [11].

Drug resistance to BRAFi induces metabolic switching from aerobic glycolysis to mitochondrial respiration and consequently increased ROS levels. Indeed, BRAF^V600E^ melanoma cells that have developed resistance to inhibitors also display increased OXPHOS, increased dependency on mitochondria for survival, increased ROS production and associated switch from glucose to glutamine metabolism [11,13,81,[98], [99], [100], [101]]. It was reported that metabolic switch of melanoma cells as a response to an acquired resistance to BRAFi is irrespective of the presence of vemurafenib.

Recent studies have also shown that antioxidant protection has a significant role in melanoma drug resistance. Melanoma cells use several metabolic pathways to avoid oxidative stress by the upregulation of transcription factors Nrf2 and Sterol Regulatory Element-Binding Protein 1 (SREBP-1), pathways: ox-PPP, serine biosynthesis and antioxidant enzymes. All of them are responsible for ROS removal and lipid peroxides detoxification by direct scavenging, and/or by supplying GSH, NADPH and CoQ10.

The elevated ROS level makes BRAFi-resistant melanoma cells more sensitive to the death that is induced by pro-oxidants [98]. Melanoma cells rewire their redox metabolism to upregulate redox capacity. It has been shown that combined histone deacetylase (HDAC) and MAPK inhibition can impede some forms of MAPK inhibitors that are resistant in melanoma [102]. Furthermore, HDAC inhibitor (HDACi), vorinostat, was shown to induce a significant DNA damage and apoptotic cell death only in the MAPK-resistant cells, but not in the drug-sensitive cells, which have a lower ROS level, through the inhibition of SLC7A11, glutamate/cysteine transporter, which imports cysteine for *de novo* GSH biosynthesis, with subsequent elevation of ROS level [103].

A study using an integrative approach through bioinformatics and flux balance analysis has shown that BRAFi-resistant melanoma has an enhanced redox capacity, involving NADPH and GSH [37]. Upregulated SLC7A11 expression, and higher predicted fluxes through GSH regenerating glutathione-disulfide reductase (GSR), suggesting an efficient maintenance of GSH. In addition, the level of serine, precursor of cysteine, was also higher in drug-resistant cells.

Consistent with other reports in melanoma cells, it was recently shown that BRAFi-resistant melanoma exhibits a strong activation of Nrf2, leading to the activation of PPP, which is involved in the regeneration of GSH and SLC7A11 expression [100]. In addition to BRAFi-resistant melanoma, the role of Nrf2 was shown in melanoma cells, which also mediates temozolomide (TMZ) resistance in melanoma cells, an DNA alkylating agent, by upregulation of GSH synthesis. Combining effect of GSH inhibitor buthionine sulfoximine (BSO) with TMZ lead to higher effect of DNA damage and cell death of melanoma [104].

Serine biosynthesis and folate cycle have been shown to play a pivotal role for melanoma survival. It was shown that all enzymes involved in serine biosynthesis are upregulated in established melanoma vemurafenib-resistant cell lines. Metotrexate, an inhibitor of folate cycle, induced sensitization of drug-resistant cells to vemurafenib [105].

Lipid peroxidation and consequent ferroptosis in melanoma cells could be used as an alternative strategy to avoid drug resistance. The role of ferroptosis in melanoma as a possible therapeutic approach to avoid drug resistance, depending on the melanoma stage differentiation, was shown by Tsoi et al. [17]. ROS-induced lipid peroxidation is prevented by Sterol Regulator Element Binding (SREBP-1), which regulates *de novo* fatty acid biosynthesis (DNFA), a survival way in melanoma cells [106]. In drug-naive cells BRAFi downregulates the processing of SREBP-1 and thereby lipogenesis. Pharmacological SREBP-1 inhibition sensitized BRAF^V600E^-mutant therapy-resistant melanoma to BRAF^V600E^ inhibitors both *in vitro* and in a pre-clinical PDX model [28]. Melanoma cells have elevated DNFA gene expression following the blocking of MAPK pathway, and DNFA expression remains higher in melanoma drug-resistant cells, compared to untreated cells. Drug-resistant cells restore DFNA to promote lipid saturation and protect melanoma from ROS damage and lipid peroxidation. DNFA pathway inhibition, whether by targeting of SREBP1, or by inhibition of DNFA enzymes, exerts potent cytotoxic effects on both drug-naïve and drug-resistant melanoma cells [41,106]. These data indicate that targeting of SREBP-1 and DNFA may offer a new strategy to overcome BRAFi resistance.

## 7 Conclusion

Emerging evidences suggests that redox homeostasis and oxidative stress is involved in the development and progression of many common cancers, including melanoma [26,43,[107], [108], [109], [110]]. As a consequence, tumor cells activate adaptive antioxidant mechanisms to keep their cellular redox state below a deadly threshold [33,111]. Thus, it has been proposed that an antioxidant blockade could be exploited for therapeutic benefits. Studies addressing the role of redox vulnerabilities in melanoma progression have been proposed [17]. New evidence demonstrates that BRAF and MEK inhibitors induce an increase in ROS in melanoma cells [11,103,112]. Commonly, oxidative stress drives the metastatic ability of melanoma cells and their resistance to therapy and, accordingly, cells from melanoma patients have adapting antioxidant mechanisms to overcome the effects of high levels of ROS [19]. Understanding of the complexity of metabolic rewiring and redox adaption during melanoma development will help to design new therapeutic strategies that struggle with melanoma metastasis and drug-resistance. Different strategies have been already proposed: by blocking of antioxidant ROS modulators (SREBP-1) and antioxidants (GSH), by increasing ROS levels (with HDACi) and by induction of ferroptosis. The idea to convert BRAF-inhibitor mediated ROS increase (oxidative stress) into a lethal weapon by inhibiting antioxidant response of these tumor cells has been suggested: BRAF-resistance melanoma will be eliminated by oxidative stress using selective drugs interfering with the melanoma antioxidant response [17]. Further investigations of redox metabolism of melanoma are needed to understand the complexity of homeostasis and its modulation.

## Author contributions

Conceptualization: LA and MMS. Data curation: LA.; writing—original draft preparation: LA.; writing—review and editing: MMS.; funding acquisition: MMS.

## Funding

This research was funded by 10.13039/501100000781European Research Council (ERC) Consolidator Grant (RENDOX-ERC-CoG 647057) and European Research Council (ERC) Proof-of-Concept (MELASTOP-PoC 963865) and 10.13039/501100005010AIRC (Associazione Italiana Ricerca sul Cancro) IG Grant 20119.

## Declaration of competing interest

The authors declare no conflict of interest.
